# The WTC Disaster and Asbestos Regulations

**DOI:** 10.1289/ehp.112-a606

**Published:** 2004-08

**Authors:** John H. Lange

**Affiliations:** Envirosafe Training and Consultants, Inc., Pittsburgh, Pennsylvania, E-mail: john.pam.lange@worldnet.att.net

Landrigan et al. (2004) reported on exposure to asbestos as a result of events involving the World Trade Center (WTC). Their results are somewhat lower than that reported by others (Lange 2004) for this unfortunate event. I reported a single asbestos bulk sample at 40% asbestos (Lange 2004), although Landrigan et al. suggested that most are in the range of 1–3%. Because there was one “high” bulk sample observed, it is likely that numerous other locations had similar “elevated” asbestos levels. Airborne exposures were also elevated for a considerable time period after the event (Lange 2004). Although measurements were reported as task-length averages (TLA), it is likely that some personal samples (Lange 2004) exceeded the U.S. Occupational Safety and Health Administration permissible exposure limit (PEL) of 0.1 fibers/cm^3^ well after the first few days of the event. For example, during 1 January 2002–11 February 2002 in the west area, the arithmetic mean exposure and an upper reported value (0.500 fibers/cm^3^-TLA) were above the PEL (Lange 2004).

Landrigan et al. (2004) also reported clearance samples as fibers per millimeter squared, which likely should be in structures per millimeter squared. It should be noted that this clearance standard has not been shown to be health based, and structures per millimeter squared cannot be equilibrated or converted to fibers per millimeter squared.

Even with evidence of higher exposure levels, on the basis of reported data (Lange 2004), it is unlikely that exposure to asbestos itself will result in any actual health effects. This is because the asbestos was mostly chrysotile (Landrigan et al. 2004) and the duration of exposure for most workers was short (Lange 2003). However, as previously reported (Lange 2001, 2002, 2004), regulatory agencies ignored their own regulations at the WTC, whereas asbestos concentrations (bulk and air) for other locations would probably trigger a regulatory response and most likely a citation with a requirement of some action plan. Thus, it appears that there are two standards to be taken from the WTC, one for agencies themselves and another for all others.
